# Modular Small Diameter Vascular Grafts with Bioactive Functionalities

**DOI:** 10.1371/journal.pone.0133632

**Published:** 2015-07-23

**Authors:** Meik Neufurth, Xiaohong Wang, Emad Tolba, Bernhard Dorweiler, Heinz C. Schröder, Thorben Link, Bärbel Diehl-Seifert, Werner E. G. Müller

**Affiliations:** 1 ERC Advanced Investigator Grant Research Group at the Institute for Physiological Chemistry, University Medical Center of the Johannes Gutenberg University, Duesbergweg 6, D-55128 Mainz, Germany; 2 Division of Vascular Surgery, Department of Cardiothoracic and Vascular Surgery, University Medical Center of the Johannes Gutenberg University, Langenbeckstraße 1, D-55131 Mainz, Germany; 3 NanotecMARIN GmbH, Duesbergweg 6, D-55128 Mainz, Germany; Michigan Technological University, UNITED STATES

## Abstract

We report the fabrication of a novel type of artificial small diameter blood vessels, termed biomimetic tissue-engineered blood vessels (bTEBV), with a modular composition. They are composed of a hydrogel scaffold consisting of two negatively charged natural polymers, alginate and a modified chitosan, *N*,*O*-carboxymethyl chitosan (*N*,*O*-CMC). Into this biologically inert scaffold two biofunctionally active biopolymers are embedded, inorganic polyphosphate (polyP) and silica, as well as gelatin which exposes the cell recognition signal, Arg-Gly-Asp (RGD). These materials can be hardened by exposure to Ca^2+^ through formation of Ca^2+^ bridges between the polyanions, alginate, *N*,*O*-CMC, and polyP (alginate-Ca^2+^-*N*,*O*-CMC-polyP). The bTEBV are formed by pressing the hydrogel through an extruder into a hardening solution, containing Ca^2+^. In this universal scaffold of the bTEBV biomaterial, polycations such as poly(l-Lys), poly(d-Lys) or a His/Gly-tagged RGD peptide (three RGD units) were incorporated, which promote the adhesion of endothelial cells to the vessel surface. The mechanical properties of the biopolymer material (alginate-Ca^2+^-*N*,*O*-CMC-polyP-silica) revealed a hardness (elastic modulus) of 475 kPa even after a short incubation period in CaCl_2_ solution. The material of the artificial vascular grafts (bTEBVs with an outer size 6 mm and 1.8 mm, and an inner diameter 4 mm and 0.8 mm, respectively) turned out to be durable in 4-week pulsatile flow experiments at an alternating pressure between 25 and 100 mbar (18.7 and 75.0 mm Hg). The burst pressure of the larger (smaller) vessels was 850 mbar (145 mbar). Incorporation of polycationic poly(l-Lys), poly(d-Lys), and especially the His/Gly-tagged RGD peptide, markedly increased the adhesion of human, umbilical vein/vascular endothelial cells, EA.HY926 cells, to the surface of the hydrogel. No significant effect of the polyP samples on the clotting of human plasma is measured. We propose that the metabolically degradable polymeric scaffold bTEBV is a promising biomaterial for future prosthetic vascular grafts.

## Introduction

The number of patients both in Europe and in USA receiving prosthetic grafts for vessel reconstruction, aneurysm repair, or hemodialysis access is steadily increasing and expected to reach over 1.8 million today [1–4]. This number will surely increase further due to the prevalence of diabetes and cardiovascular diseases in the developed countries, but also and especially in the emerging economies, e.g. China and India [5]. However, until recently only limited clinical success for prosthetic vascular grafts has been achieved. The annual costs for those grafts increase to over 25 billion € in Europe, where prosthetic vascular grafts are primarily applied/sold as endovascular stent-grafts for aortic aneurysm repair (nearly 87%), followed by peripheral vascular grafts (10%), and grafts for hemodialysis access (3%) or small-diameter coronary artery bypass surgery (<1%). Without any question, autologous vessels (donor and recipient are the same person) are the preferred grafts for reconstruction/substitution of damaged vascular segments with inner diameters of < 5 mm [3]. Since many patients, due to comorbid conditions or previous interventions, lack autologous vessels suitable for a surgical transfer an urgent clinical need for prosthetic substitutes that can compete with autologous vessels exists.

A suitable vascular graft should be readily available (ideally off-the shelf), being durable during long-time implantation, not eliciting an inflammatory potential, and not promoting thrombosis and/or infection [6]. In addition, the graft wall should have similar mechanical properties like the native host vessel [7]. At present, the most frequently applied materials for synthetic vascular grafts are made of expanded polytetrafluroethylene (ePTFE) and of polyethylene terephthalate (PET) [8]. Both materials show sufficient long-term results for large-scale arterial reconstruction and large-diameter vessels (> 6 mm) but have been proven to display inferior performance and biological properties in small-diameter applications [9]. The failure in the latter applications of these synthetic implants are caused by an increased surface thrombogenicity, due to the lack of a functional endothelium, and a substantial development of intimal hyperplasia as a consequence of chronic inflammations [10,11]. A severe consequence of the lack of a functional endothelial layer is an increased microbial contamination, which often needs implant replacement. In order to comply with the native tissue environment intensive attempts are ongoing to develop artificial or bioartificial vascular grafts that do not include harvesting of cells, followed by pre-culture procedure [12]. Those implants should allow the formation of an endothelial cell (EC) layer in the lumen of the prosthetic vessel grafts, which prevents platelet aggregation and smooth muscle cell hyperproliferation. In turn, the initial step of endothelialization should be addressed as the critical event in the development of vascular prostheses in order to avoid graft reocclusion [13]. Consequently, synthetic substitutes should allow *in vivo* graft endothelialization, involving EC migration from graft anastomoses or by endothelial progenitor cells *via* tuned cell adhesion and migration. Previously collagen-composed vascular grafts have been proposed to support *in vivo* graft endothelialization; however, this approach had been abandoned because native collagen is thrombogenic and in addition those grafts lack the required mechanical properties of a functional vessel [14].

At present small diameter vascular grafts with an internal diameter of less than 5 mm are not approved by the FDA for clinical use due to their high failure rates [15]. The major reason is that those grafts have the potential to promote thrombosis [16] as well as to enhance endothelial cell proliferation [17]. First attempts to overcome this problem included the introduction of bioengineered graft materials, using polyurethane [17] or silk [16] as a scaffold. In our approach, presented here, we introduce novel small diameter vascular grafts with different bioactive functionalities. Such grafts, biomimetic tissue-engineered blood vessels (bTEBV), which should meet the individual needs of a given patient, can be fabricated in a modular way, by using a scaffold backbone, supplemented with biologically active polymers, “embedded biofunctionally active polymers” that are linked with biofunctionally active ligands. In the present study we describe the fabrication of bTEBV built from a “universal, inert scaffold” into which “biofunctionally active polymers” have been incorporated.

Universal, inert scaffold: In order to circumvent the problems associated with the use of animal-derived collagen, synthetic polymer scaffolds have been fabricated that are supplemented with extracellular-matrix (ECM)-derived peptides [2]. For the latter study a poly(ethylene glycol) (PEG) hydrogel has been formulated that allows the fabrication of vessels that are composed of several layers. In our approach we have developed a new hydrogel that is built, as a backbone, by two natural polymers, first alginate, a natural plant polymer, composed of unbranched chains of (1,4) linked β-d-mannuronate and α-l-guluronate residues which are arranged in a blockwise fashion [18] and second, a modified crab chitosan that is produced, after deacetylation, from chitin, the second most abundant biopolymer [19]. The initially present units in chitosan, the β-(1–4)-linked d-glucosamine and *N*-acetyl-d-glucosamine, were converted to carboxymethyl groups, allowing, like alginate, the binding to cations; it is termed *N*,*O*-carboxymethyl chitosan (*N*,*O*-CMC) [20]. The characteristic feature of the biomaterial, synthesized by us, is that the two polymers, alginate and *N*,*O*-CMC, can be fabricated to a bioprintable and biospinnable soft hydrogel that can be hardened *via* formation of Ca^2+^ bridges (alginate-Ca^2+^-*N*,*O*-CMC) to a durable hydrogel [21,22].

The mechanical properties of the newly developed biomaterial (alginate-Ca^2+^-*N*,*O*-CMC), the hardness, can be tuned and easily adjusted. In turn, this material is qualified to be used, in a suitable manner, with respect to the topological and spatial circumstances, in an age-adapted, customized way. It is well established that the mechanical properties of the blood vessels, the arteries and veins, drastically change age-dependently and in various disease conditions [23]. The elasticity/stretchiness and stiffness of the blood vessels are dependent on a series of factors and have multiple causes and locations [24], which are not only determined by structural, cellular, and genetic factors but also by degenerative, pathophysiological changes of the scaffold proteins, in the extracellular matrix, of inflammatory molecules or the function of the endothelial cells. Therefore, the proper selection of the vascular graft, matching the mechanical, viscoelastic properties of the adjacent arterial or venous vessels is decisive for the success of the implantation.

The material studied in the present investigation is durable. As outlined, the vascular grafts can be functionally employed with a pulsatile, rhythmic flow for over 4 weeks without leakage.

Embedded biofunctionally active polymers: The basic scaffold applied here, alginate-Ca^2+^-*N*,*O*-CMC, is biologically inert, as shown for alginate [25] and the chitosan-derivative [26]. As a first component, with biological activity which has been shown to be suitable for addition to the alginate [27] or alginate/chitosan hydrogel [21,22], gelatin is added. This natural polymer, processed from collagen, retains the property to bind to integrins on the attaching cell surfaces. Gelatin exposes the Arg-Gly-Asp (RGD) cell recognition signal [28,29] and, by that, allows the cell-membrane bound integrins to bind [30,31].

A polymer of choice to be embedded into the universal scaffold is polyphosphate (polyP) that, like alginate and *N*,*O*-CMC, is exposing negatively charged groups (reviewed in: [32]). PolyP is synthesized by platelets and secreted after activation [33,34]; in addition it is released by infecting bacteria [32]. PolyP has been described as a key modulator in platelet-mediated pro-inflammatory and pro-coagulant disorders [34]; however these data are controversial [35]. The authors were using a polyP purification procedure based on an ion-exchanger [36] which yields in the eluate polyP as a Na^+^ salt. Likewise studies with activated mast cells have been performed with Na^+^-polyP [37]. As the authors discussed, based on their *in vitro* studies, this effect might be attributed to binding of key elements of the respective pathways to anionic surfaces, resulting e.g. in a conformational change in the FXII zymogen. In serum/plasma a total Ca^2+^ concentration of ≈2 mM exists [38] that is enough to replace Na^+^ by Ca^2+^ in the soluble polyP, whereby polyP becomes insoluble [32]. Also to add that polyP, as a Na^+^-polyP salt, readily undergoes hydrolysis to monomeric phosphate *via* alkaline phosphatase (ALP) [39] which is highly abundant in the extracellular environment of the endothelial cells [40]. In turn, we co-added polyP (as Na^+^-polyP) to the alginate and *N*,*O*-CMC during the preparation and the subsequent form/pattern printing [27] and subsequently exposed the material to Ca^2+^ to harden the material and simultaneously abolish the anionic properties of the polymers [21,22]. In bone-like SaOS-2 cells as well as on mesenchymal stem (MSC) cells, polyP has been described to display a mineralization inducing function and induces gene expression of the *bone morphogenetic protein-2* (BMP-2) and the enzyme *ALP* and as well as of *collagen type I* [41,42]. This finding has recently been confirmed and extended *in vitro* [43] and *in vivo* [44]. A series of other mammalian cells, e.g. H1299 (human non-small cell lung carcinoma cells), U251 (human glioma cells) and HEK293 (human embryonic kidney cells), have been found to show an increased proliferation rate in response to polyP exposure [45]. PolyP is known to induce in MSC, especially during differentiation to osteoblasts, morphogenetic factors or enzymes, e.g. BMP-2 and alkaline phosphatase [42]. The molecular mechanism by which polyP affects MSC is not yet studied; a potential candidate pathway is the target of rapamycin (TOR)-kinase signaling, which is involved in proliferation and differentiation of human cells [46]. Likewise important is the fact that the formation of endothelial tissue during vasculogenesis is a process during which the embryonal angioblasts differentiate from mesodermal cells/MSCs [47] under an organization of a primordial vascular network occurs [48]. Likewise anabolically influencing the cell’s metabolism is biosilica, a natural polymer that causes, especially in bone-(related cells), an inducing effect on cytokines, e.g. BMP-2 [49].

The scaffold backbone of the bTEBV, hierarchically structured from the above mentioned polymers, all of them are (poly)anionic, is hardened/linked together by cationic Ca^2+^ to cylinders and discs, displaying a stiffness and a hardness even suitable for use as a bone implant [21,22]. In the approach summarized here also other bi- to polyvalent cations, e.g. the polycationic poly-l-lysine or poly-d-lysine, can be incorporated into the scaffold. Those oligo/polymers which support the attachment of cells to the EM, as shown for small intestinal crypt cells IEC-6 cells [50] and endothelial cells [51,52] increase the cell attachment to natural and artificial surfaces followed by an activation of cell metabolism. In the present study we also incorporated into the bTEBV scaffold the peptide NH_2_-RGDGGRGDGGGRGDGGGHHHHHH-COOH (termed His/Gly-tagged RGD) which contains three copies of the Arg-Gly-Asp (RGD) cell recognition signal, in order to increase the propensity of the endothelial cells to adhere to the artificial vessel surface. The amino acid His has been included into the peptide in order to utilize the property of this amino acid to form weak interactions with alginate [53] and to bind to negatively charged bio-polymer surfaces [54].

**Fig 1:**
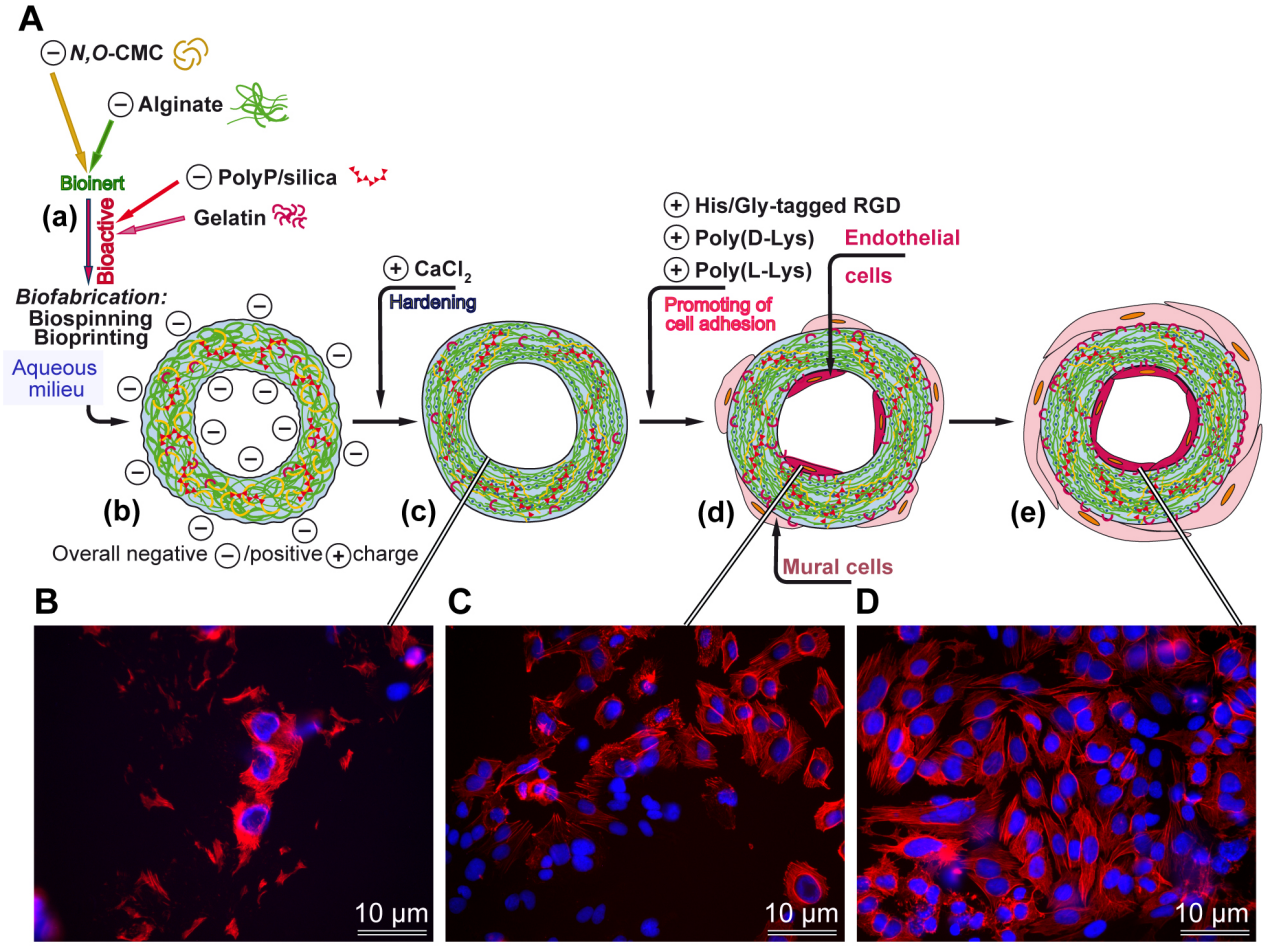
Fabrication of biomimetic tissue-engineered blood vessels (bTEBV). (**A**) The scaffold is composed of the negatively charged inert polymers, *N*,*O*-carboxymethyl chitosan (*N*,*O*-CMC) and alginate. This scaffold is enriched with the bioactive polyanionic polyP, as well as gelatin. The hydrogel is pressed through an extruder and immediately submersed in a solution, containing the cation Ca^2+^, followed by hardening of the material. The Ca^2+^ can be partially substituted by polycations, e.g. poly(l-Lys), or the peptide His/Gly-tagged RGD. After addition of poly(l-Lys), especially together with the His/Gly-tagged RGD peptide, the endothelial cells densely cover the surface of the hydrogel. The outer cell layer, composed of mural cells, is only sketched for completeness, but not studied here. (**B** to **D**) Human, umbilical vein/vascular endothelial cells, EA.HY926 cells, grown for 2 weeks on the following scaffold matrices; (B) basic scaffold alone, (C) basic scaffold, supplemented with poly(d-Lys), or (C) basic scaffold, supplemented with His/Gly-tagged RGD. The cells are stained, after fixation, with DRAQ5 (blue fluorescence) and labeled antibodies against actin (red), as described under “Material and methods”.

A scheme of the composition of the developed potential prosthetic vessel is given in Fig 1A; it outlines the functional arrangement and composition of the components, composing the prosthetic vascular graft: scaffold backbone formed by the inert anionic polymers, *N*,*O*-CMC and alginate, enclosing the embedded biofunctionally active polymers, anionic polyP and gelatin, that are linked together *via* the divalent cations. Incorporated into this scaffold have also been poly(l-Lys), poly(d-Lys) and/or the His/Gly-tagged RGD peptide in order promote the adhesion of the endothelial cells to the material. The properties, summarized in the present study, might qualify the use of this biomaterial for the fabrication of bTEBV to be used in preclinical, animal studies.

## Material and Methods

### Materials

Sodium polyphosphate (Na-polyP of an average chain of 40 phosphate units) was obtained from Chemische Fabrik Budenheim (Budenheim; Germany); sodium silicate solution (338443) from Sigma-Aldrich (Steinheim; Germany). The peptide NH_2_-RGDGGRGDGGGRGDGGGHHHHHH-COOH (termed His/Gly-tagged RGD) had been chemically synthesized by Coring System Diagnostix (Gernsheim; Germany), applying the solid-phase peptide synthesis technique and using the Fmoc strategy [55].

### Preparation of *N,O*-carboxymethyl chitosan

*N*,*O*-carboxymethyl chitosan (*N*,*O*-CMC) was prepared from chitosan (from shrimp shells, C3646; Sigma-Aldrich) as described [56–58] and sterilized with a 12-W ultraviolet lamp (Syngene, Cambridge; UK) at 254-nm wavelength (distance of 10 cm; 12 h). A suspension of 50 mg/ml of *N*,*O*-CMC, in sterilized physiological saline (0.9% [w/v] NaCl), supplemented with 30 μM silica (prepared from Na-silicate), was prepared and stirred until it became homogeneous. Then 20 mg/ml of solid Na-polyP was added and again stirred until a close to uniform solution was reached. The resulting hydrogel solution was supplemented with 50 mg/ml of sodium alginate (W201502; Sigma) and brought to homogeneity at 50°C, while stirring. If not mentioned otherwise this hydrogel, the “basic scaffold” was enriched with 0.1% low-melting gelatin (bovine; 22151.02; SERVA, Heidelberg; Germany) that was added together with the alginate.

### Addition of polycations and His/Gly-tagged RGD

It is crucial that a scaffold used to fabricate bTEBV provides a suitable template for the cells to adhere to [59]. In our studies we added to the basic scaffold the following components at a concentration of 3 μg/ml: poly(l-Lys) (as hydrobromide; mol wt 70,000–150,000; P6282, Sigma), poly(d-Lys) (as hydrobromide; mol wt 70,000–150,000; P6407, Sigma) or His/Gly-tagged RGD. Poly(l-Lys) and poly(d-Lys) were added to the scaffold after hardening with CaCl_2_, while His/Gly-tagged RGD was added prior to the crosslinking of the basic scaffold components (*N*,*O*-CMC, polyP, alginate and gelatin). Subsequently the material was extruded or printed and finally hardened as described. The material is termed “poly(l-Lys)-scaffold”, “poly(d-Lys)-scaffold” or “RGD-scaffold”.

### Fabrication of bTEBV: Extruder

**Fig 2:**
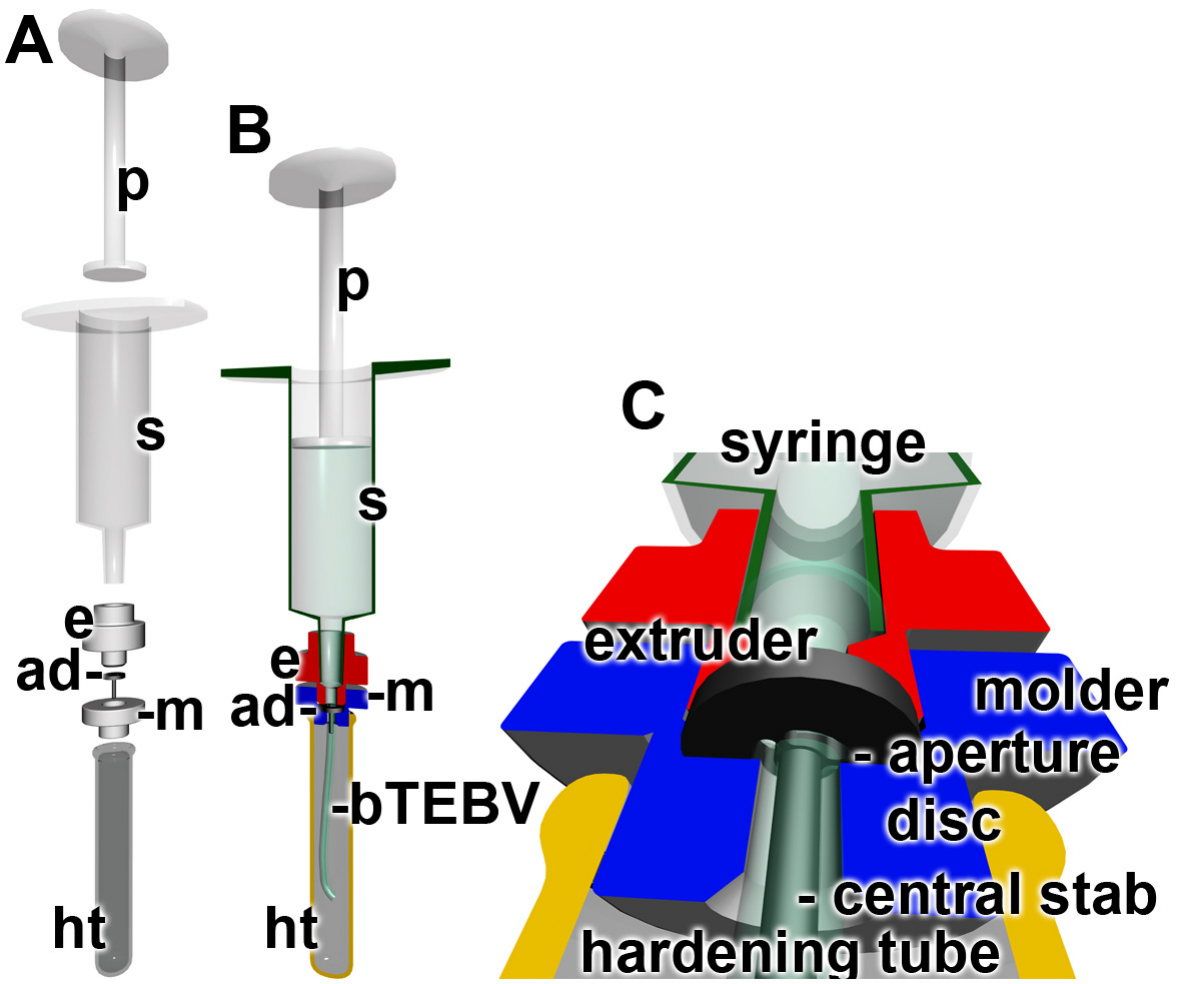
Technical drawing of the extruder used for the fabrication of the bTEBV. The *N*,*O*-CMC-based material is filled into a syringe (s) which is hooked to the extruder (e). (**A** and **B**) By pressing the material with the plunger (p) into the syringe and through the aperture disc (ad), hooked within the molder (m) via the aperture disc in the molder, into the hardening tube, a tube around the central stab is formed. The tube, the bTEBV, undergoes hardening in the hardening tube, a process driven by Ca^2+^.

Biomimetic tissue-engineered blood vessels (bTEBV) were fabricated by an extruder through which the *N*,*O*-CMC/alginate/polyP/gelatin hydrogel was continuously pressed through a nozzle (Fig 2). The hydrogel was filled into a sterile 5 ml syringe (Discardit II, BD Heidelberg; Germany). Prior to the extrusion process to fabricate the vessels, the syringe was centrifuged (in direction of the gravity) to remove the remaining air bubbles (3 min; 1500 rpm). Then the plunger was inserted and the filled syringe was stored at 4°C until use. At the beginning of the squeezing out step of the bTEBV material, the temperature of the syringe was brought to 20°C (30 min). The syringe was connected to the home-made extruder unit which contained a laser-cut stainless steel aperture disc. Three curved openings separated by 200 μm wide partitions were arranged in this disc in a circular way in order to allow the passage of the hydrogel. The three hydrogel strands immediately fuse together after their release around a central stab. Variation of the distance of the center of the central stab to the outer edge of the outlets of the laser-cut stainless steel aperture disc as well as variation of the diameter of the central stab allows the fabrication of vessels with an outer diameter from 1.8 mm (comprising a central tube diameter of 0.8 mm) to 6 mm (tube inner diameter of 4 mm); vessels with an intermediate size of 2.95 mm (0.95 mm) have also been produced. The tubes were immediately submersed into a 2.5% [w/v] aqueous CaCl_2_ solution. During a (routinely) 5 min incubation period the vessels formed acquired their final hardness. Prior to the experiments the ends of the vessels were clipped off; usually 6 cm long vessels were made.

In order to increase the hardness of the vessels the hardening procedure of the bTEBV was performed in a 2.5% [w/v] aqueous CaCl_2_ solution, supplemented with 70% [v/v] ethanol. Under those conditions the duration of the hardening could be shortened to 3 min (in maximum).

### Bioprinting of gel discs

Discs with the material used for the testing of adhesion and growth of endothelial cells were bioprinted. The procedure for printing was performed essentially as recently described [27]. The respective hydrogel preparation was filled into sterile 30 ml printing cartridges (Nordson EFD, Pforzheim; Germany) and centrifuged for 3 min at 1500 rpm to remove air bubbles. After connecting a 0.25 mm tapered polyethylene printing tip (Nordson EFD) the cartridge was placed into the preheated (25°C) printing head of the 3D-Bioplotter (Envisiontec, Gladbeck; Germany). At 25°C, using a pressure of 1.5 bar and a printing speed of 16 mm/s cylindrical scaffolds measuring 7.5 x 0.4 mm were printed as described [27]. The strand distance between the printed cylinders was set to 1 mm resulting in a pore size of the printed layers of approximately 0.5 x 0.5 mm. Those scaffolds were printed directly into sterile 94 mm Petri dishes (Greiner Bio-One, Frickenhausen; Germany), supplemented with 2.5% [w/v] CaCl_2_ as crosslinking solution. The printed discs with a diameter 9.5 mm had been hardened in 2.5% [w/v] CaCl_2_ for crosslinking for 5 min. During this treatment the discs shrunk to 7.5 mm.

### Burst pressure experiments

The studies to determine the burst pressure of the fabricated bTEBV were performed [60] using tubes of a size of 1.8 mm (inner diameter: 0.8 mm) and 6 mm (4 mm). Those tubes were pulled on both ends over either FEP (fluorinated ethylene propylene) hoses from intravenous catheters, measuring 0.9 mm outer diameter or 4 mm luer-hose-connectors (Carl Roth GmbH, Karlsruhe; Germany). The hoses were inserted approx. 10–20 mm deep into the lumen of the bTEBVs. This setup was connected to a pump system (BioRad Econo Pump; BioRad, München; Germany) with an attached pressure gauge. The liquid reservoir was placed into a water bath at 37°C to mimic body temperature. For routine experiments the pump was adjusted to 3–6 ml/min and the pressure was regulated between 25–100 mbar using a hose clamp, mounted downstream of the sample. For the determination of the burst pressure, the liquid flow was totally blocked downstream of the sample. The pressure was then steadily increased until the sample showed severe leakage or complete burst.

### Elastic modulus, reduced Young's modulus, of the biomaterial

As a parameter for the elastic modulus the hardness of the *N*,*O*-CMC-polyP scaffold was determined, using a ferruled optical fiber-based nanoindenter as described [61,62]. The indents were depth controlled (10 μm) and the loading and unloading period was set to 2 s. Based on the load-displacement curves the reduced Young's modulus (RedYM) was calculated [63].

### Cell culture

As a model for endothelial cells, human umbilical vein/vascular endothelial cells, the transformed EA.HY926 cells ATCC_CRL-2922 [59,64] were used. The EA.hy926 cell line is a hybrid between HUVEV cells with the human lung carcinoma cell line A549. The cells were grown in Dulbecco's Modified Eagle's Medium (DMEM; low glucose; D5921 Sigma), supplemented with 10% fetal bovine serum (FBS), 50 μg/ml gentamycin (G1397 Sigma), 2 mM glutamine (K0282; Biochrom, Berlin; Germany) and 2% HAT Media Supplement (H0262 Sigma). The cells were layered onto the printed gel discs at a density of 40,000 cells/well (= 500 μl) in 48 well plates (0.95 cm^2^ growth area; Greiner, Frickenhausen; Germany). Splitting of the medium/serum was performed twice in a week.

Growth of the cells, after an incubation period of 7 d, was determined by a colorimetric method based on the tetrazolium salt XTT (Cell Proliferation Kit II; Roche, Mannheim; Germany), according to the recommendations of the supplier and as described [65]. The printed discs with a diameter 0.95 cm were hardened in 2.5% [w/v] CaCl_2_ for crosslinking for 5 min. The following discs were used for the experiments: “control-scaffold”, containing none of the adhesion promoting polymers poly(l-Lys), poly(d-Lys) or RGD-scaffold, “poly(l-Lys)-scaffold”, “poly(d-Lys)-scaffold” or “RGD-scaffold”. A 500 μl cell suspension (80,000 cells/ml) was added per well and incubation was continued for 7 d, followed by determination of viability using the XTT assay.

### Staining of cells

The cells attached to the surface of the scaffold were fixed in formaldehyde vapor [66], then permeabilized in PBS (phosphate buffered saline), containing 0.5% (v/v) Triton X-100 (T8787 Sigma) for 1 h (room temperature). To visualize the cells, they were stained with rhodamine phalloidin (PHDR1; Biomol, Hamburg; Germany) as well as DRAQ5 (Biostatus Ltd., Shepshed; UK; nuclear stain), as described [67]. The actin complexes were visualized at an excitation of 550 nm and an emission of 570 nm, while for detection of the DRAQ5 fluorescence the cells were excited at 635 nm, and recorded at an emission of 705 nm.

### Determination of the clotting time

The established method was applied [68]. Normal human citrated plasma (P9523 Sigma) was mixed with 100 μM of prewarmed polyP, complexed with Ca^2+^ in a stoichiometric molar ratio of 2 (with respect to phosphate monomer) to 1 Ca^2+^ [41], or 10 μg/ml of beads formed of *N*,*O*-CMC, and Na-polyP (as outlined above) and hardened with 2.5% [w/v] aqueous CaCl_2_ solution [49] were added at 37°C in coagulometer cuvettes; the measurements had been performed in a Thrombotrack 4 coagulometer (Diagnostica Skalpeli, Zagreb; Croatia). After addition of CaCl_2_ [69] the clotting time was determined. Ten parallel experiments had been performed and the means (± S.D.) are given.

### Reverse transcription-quantitative real-time PCR analyses

The technique of reverse transcription-quantitative real-time polymerase chain reaction (RT-qPCR) was applied to determine the gene expression level for *CD31* in the EA.HY926 cells. The experiments were performed technically as described [70]. The cells were incubated in medium/serum for 9 d onto “control-scaffold”, “poly(d-Lys)-scaffold”, “poly(l-Lys)-scaffold” and “RGD-scaffold”. Then the cells were collected and the RNA was isolated and subjected to RT-qPCR. The following primer pair was used for the determination of the expression of *CD31* gene Fwd: 5’-TCCCCTAAGAATTGCTGCCA-3’ and Rev: 5’-TTCTTCCCAACACGCCAATG-3'; as reference gene *GAPDH* (glyceraldehyde 3-phosphate dehydrogenase) was applied with the following primer pair Fwd: 5′-CCGTCTAGAAAAACCTGCC-3′ and Rev: 5′-GCCAAATTCGTTGTCATACC-3′. The amplification had been performed in an iCycler (Bio-Rad, Hercules, CA; USA) with the respective iCycler software. After determinations of the C_t_ values the expression of the respective transcripts were calculated [71]. The expression level of the *CD31* gene was determined; the expression level of the *CD31* had been normalized to the one of the reference gene *GAPDH*.

### Light microscopic analyses

Digital light microscopic studies were performed using a VHX-600 Digital Microscope (Keyence, Neu-Isenburg; Germany) equipped with a VH-Z25 zoom lens. The fluorescence of the cells was excited at 635 nm, and the emission was recorded at 705 nm in an Olympus IX71 fluorescence microscope.

### Statistical analysis

The results were statistically evaluated using paired Student’s *t*-test [72].

## Results

### Preparation of the *N,O*-carboxymethyl chitosan-based scaffold

**Fig 3:**
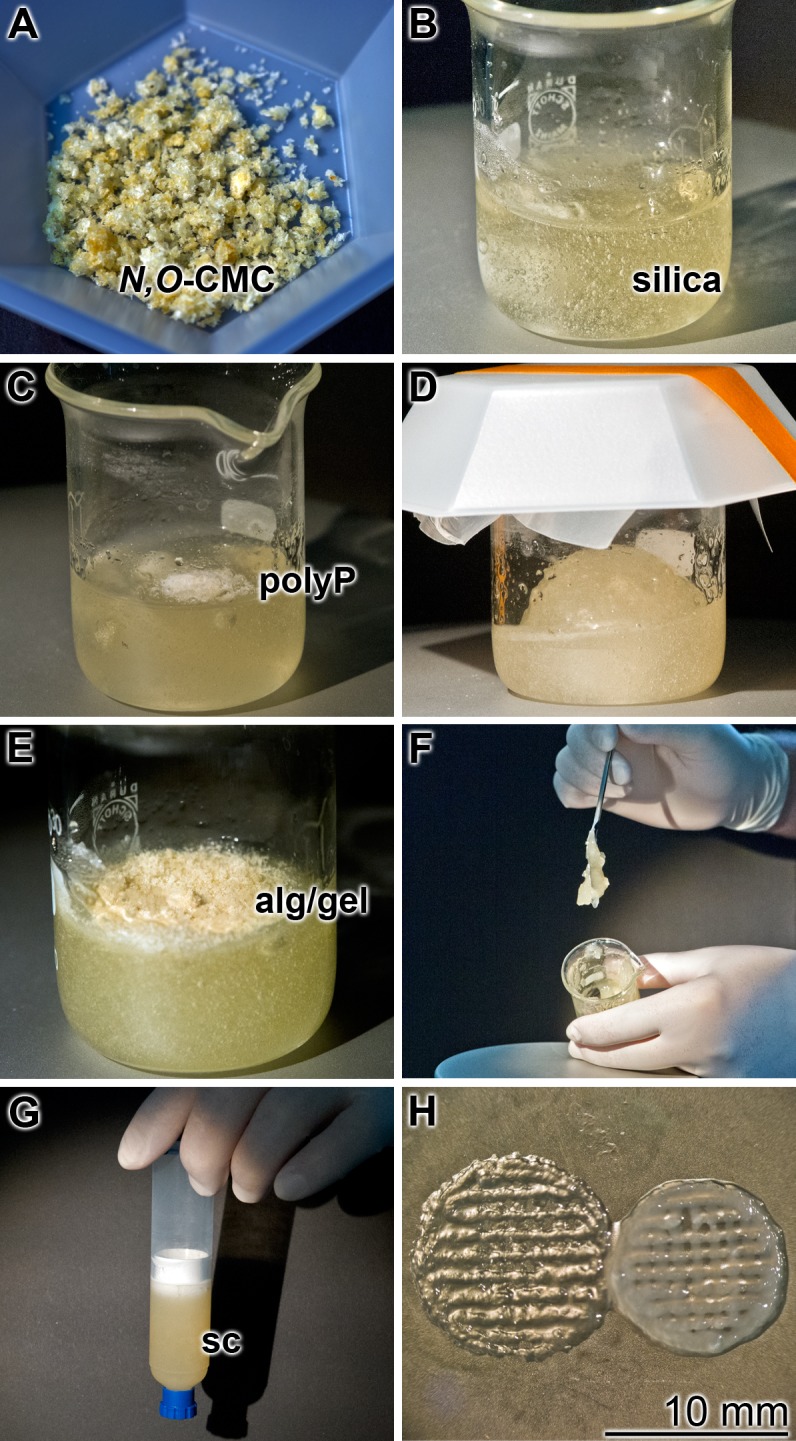
Sequential addition of the polymers to prepare the hydrogel. (**A** and **B**) Suspending of *N*,*O*-CMC and silica in saline, and preparation of a solution; (**C** and **D**) addition of Na-polyP (polyP) and preparation of a homogeneous solution, again by stirring; (**E** and **F**) supplementation of solid sodium alginate (alg), together with low-melting gelatin (gel), resulting in a viscous gel. (**G**) Filling of the hydrogel, the scaffold to be used for fabrication of the bTEBV, in the extruder or for bioprinting, into a syringe. (**H**) Printed hydrogel discs, prior (left) or after (right) hardening with CaCl_2_.

The **universal scaffold** was prepared from *N*,*O*-CMC, silica, polyP, alginate/gelatin in a fixed sequence, as outlined under “Material and methods” (Fig 3). The hydrogel was prepared from the biologically inert polyanionic polymers, *N*,*O*-carboxymethyl chitosan (*N*,*O*-CMC) and silica, suspended in saline (Fig 3A and 3B). During stirring a homogeneous, viscous solution of these anionic polymers was obtained that was supplemented with the bioactive, anionic, polymers polyP, silica and gelatin, as outlined under “Material and methods”. The sequence of addition of the components is crucial (Fig 3). At first *N*,*O*-CMC and silica are suspended in saline and then stirred until homogeneity (Fig 3A and 3B). In the second step solid Na-polyP is added which can be brought to a homogeneous gel (Fig 3C and 3D). In the third step solid sodium alginate together with low-melting gelatin is added, resulting in a highly viscous gel (Fig 3E and 3F). This hydrogel is then filled into the syringe (Fig 3G) that is fitted with its plunger to the home-made extruder, to fabricate the vessel or in the bioprinter, to print the gel discs (Fig 3H), after centrifugation (removal of air bubbles).

The **biofunctionally active polymers** were embedded into the scaffold either during its preparation (His/Gly-tagged RGD) of after completion and hardening of the scaffold (poly(l-Lys) and poly(d-Lys)).

Accordingly, three types of scaffolds were prepared; “control scaffold” containing *N*,*O*-CMC, silica, polyP and gelatin, and scaffolds enriched with the active polymers poly(l-Lys), poly(d-Lys) and His/Gly-tagged RGD that are termed “poly(l-Lys)-scaffold”, “poly(d-Lys)-scaffold”, and “RGD-scaffold”.

### Fabrication of the bTEBV

**Fig 4:**
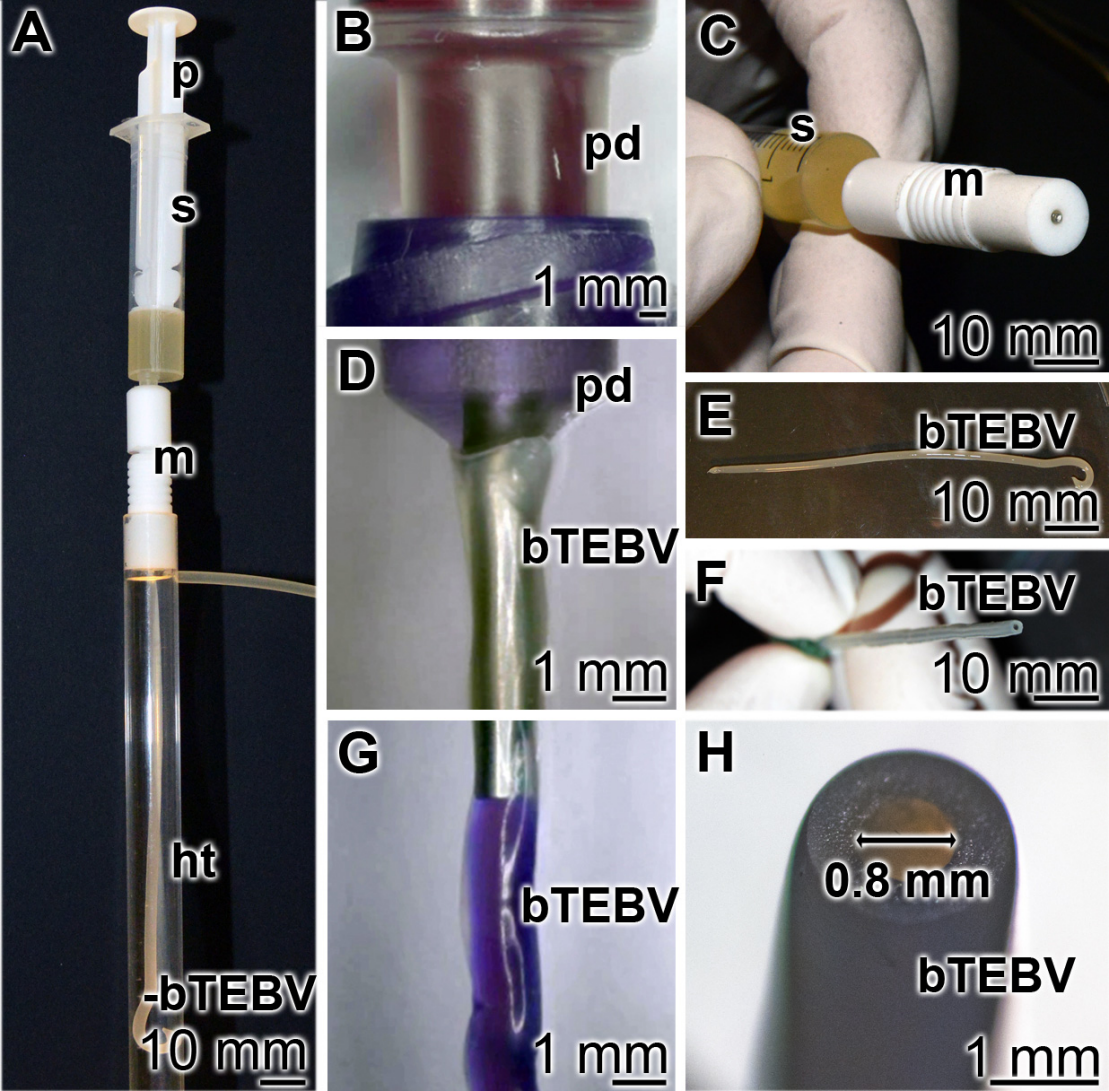
Experimental fabrication of the bTEBV in the home-made extruder. (**A**) The extruder devise with its main parts, the plunger (p), syringe (s), molder (m) and the prepared bTEBV in the hardening tube (ht) is shown. (**B**, **D**, **G**) The ready fabricated bTEBV is pulled over a tube outlet of the pressure device (pd). In (G) the cell culture medium that has been pumped into the vessels is colored in purple. Close up of the (**C**) central part of the extruder, with the syringe (s) and the molder (m), and (**E**, **F**, **H**) the finally fabricated bTEBVs with an inner diameter of 0.8 mm are depicted.

The scaffold material was pressed through the extruder (Fig 4). After passage of the material through the molder and submersing the extruded bTEBV in the hardening tube, filled with 2.5% [w/v] aqueous CaCl_2_ solution (Fig 4A), the bTEBV can be taken out and pulled over a stainless steel tube outlet (Fig 4B, 4D and 4G). The test solution (here stained in purple; Fig 4G) is then squeezed *via* the pressure syringe into the bTEBV. A close-up of the extruder with its central molder (Fig 4C), as well as of the readily fabricated bTEBVs, here with an inner diameter of 0.8 mm, is also shown (Fig 4E, 4F and 4G).

**Fig 5:**
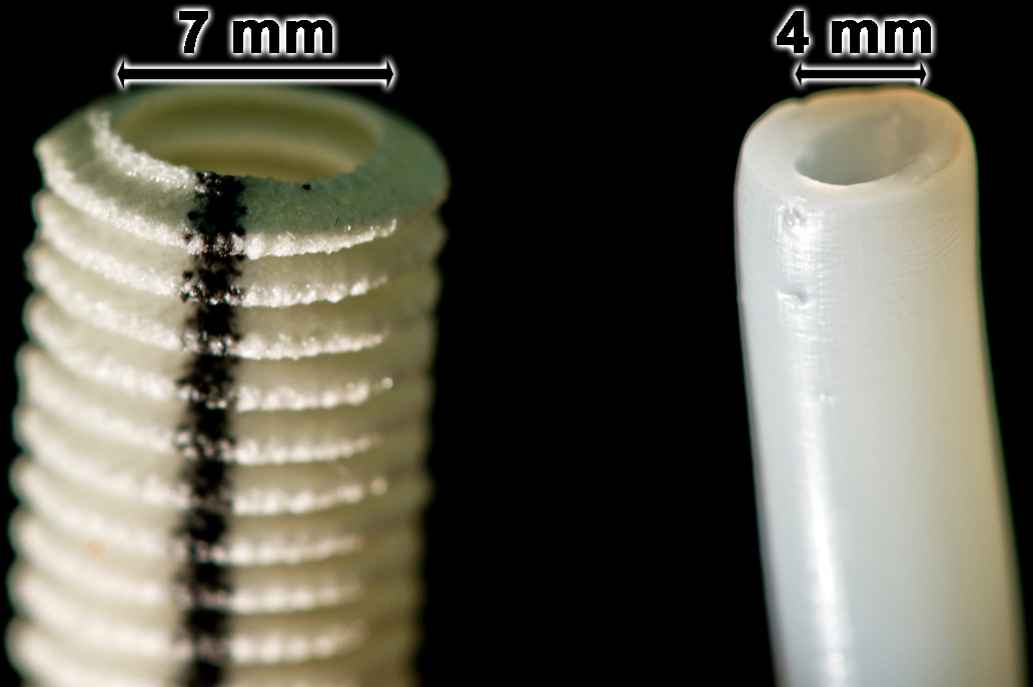
Inner dimension of the bTEBV. Changing the aperture disk within the extruder housing from the one with a 0.8 mm central stab resulting in a smaller material extrusion to aperture discs with a 4 mm central stab as well as equally enlarged material outlets allows the fabrication of bTEBV with an inner diameter of 4 mm (**right vessel**) instead of the 0.8 mm sized bTEBV (inner diameter). **Left vessel**: commercially available vessel grafts, totally prepared from non-biological polymers.

Through variation of the distance of the center of the central stab to the outer edge of the outlets of the laser-cut stainless steel aperture disc, or through variation of the diameter of the central stab vessels with an outer diameter of 6 mm (inner diameter, 4 mm) (see Fig 5 right; for comparison, a commercial synthetic vessel graft is shown on the left) or 1.8 mm (inner diameter of 0.8 mm), different dimensioned vessells have been produced.

### Mechanical characterizing of the vascular grafts

The extruded bTEBV were fabricated either with a diameter of 6 mm or 1.8 mm (outer diameter), and an inner diameter of 4 mm or 0.8 mm, respectively. During the hardening process shrinkage of the biomaterial occurred which leads to slightly reduced diameter of the vessels. In order to underscore the uniformity of the bTEBV the outer and inner diameters as well as the wall thicknesses along a 6 cm long vessel was measured in sequential intervals of 5 mm. These determinations revealed that larger vessels (6 mm) comprise a mean outer diameter along the bTEBV of 5.62±0.08 mm (n = 12) and an average inner diameter of 4.1±0.07 mm (n = 12). Due to the shrinkage of the biomaterial a reduction of the outer diameter (by 6.7%) and a slightly increase of the inner diameter (2.5%) appeared. A similar result was obtained with the smaller vessels (1.8 mm); a mean outer diameter of 1.65±0.03 mm (n = 12) and a mean inner diameter of 0.76±0.01 mm (n = 12) is seen. These data reflect a reduction of the outer diameter by 8.2% and of the inner diameter by 5.6%. The wall thickness was measured on each section of the inspected vessels as triplicates. In case of the large vessels the data show a mean wall thickness of 735±35 μm (n = 12), while the small vessels show a mean wall thickness of 448±25 μm (n = 12).

Addition of the bioactive components Poly(l-Lys) and poly(d-Lys) did not change those parameters significantly.

### Determination of the burst pressure of the bTEBV

**Fig 6:**
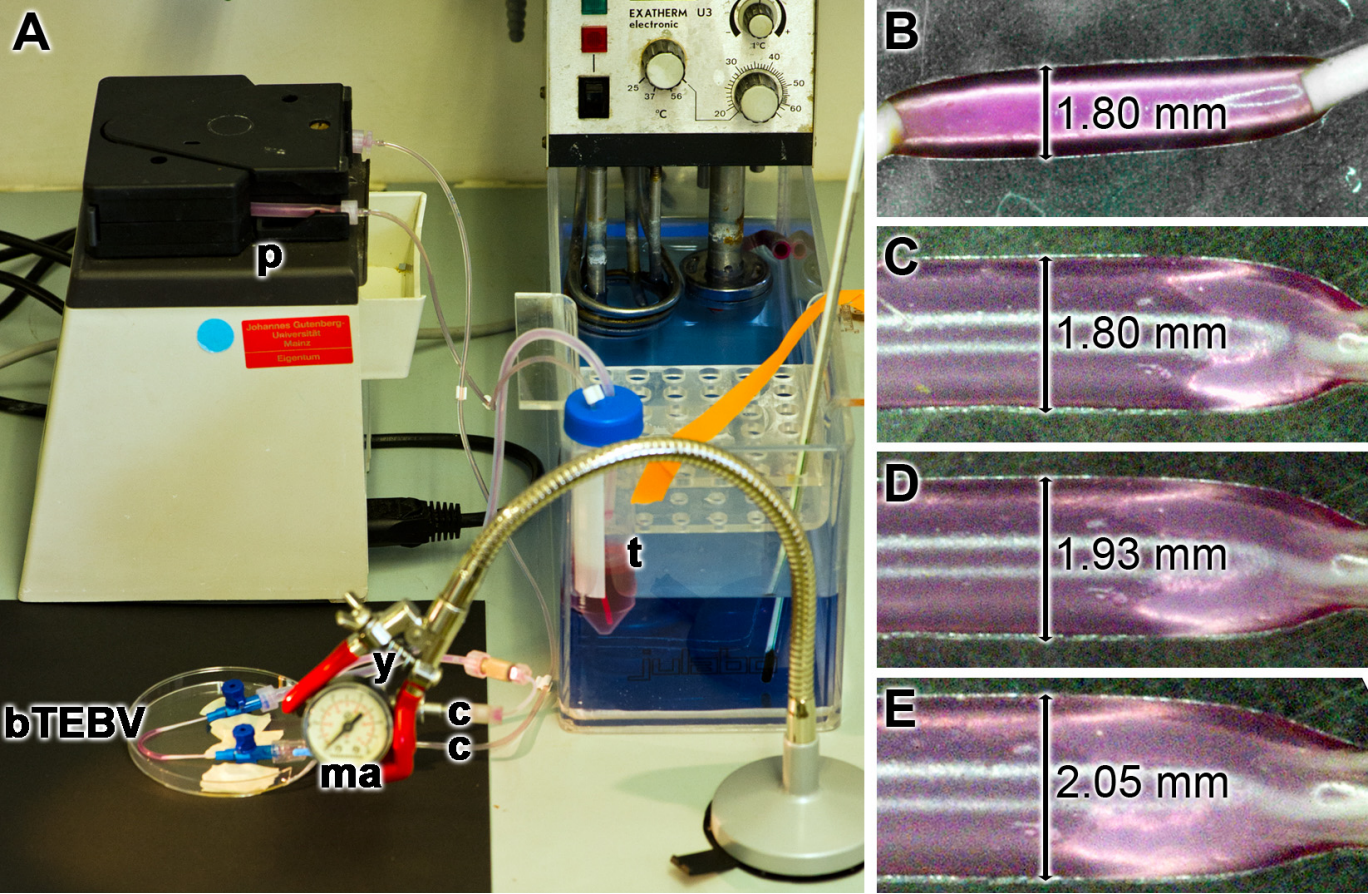
Pressure testing of the bTEBV. Here the small sized vessels, 1.8 mm (inner diameter 0.8 mm) were examined. (**A**) Device for studying the stability of the vessels. The two ends of the respective bTEBV were connected *via* hoses to the connecting tubes (c) that are in full close with the pump (p). The manometer (ma) was connected with the water flow by a Y connection (y). Colored (purple) medium was alternatively pumped into the vessels; it was temperature-controlled at 37°C, using a thermostat (t). (**B** to **E**) During the pumping cycle the outer diameter of the vessels varies between 1.80 mm and 2.05 mm.

Human muscular arteries vary in size from an outer diameter of ≈ 10 mm to about 0.5 mm (mean diameter ≈ 4 mm and mean wall thickness ≈ 1 mm), while the similarly sized veins have wider inner diameters, with a mean diameter of ≈ 5 mm and a mean wall thickness of ≈ 0.5 mm. Therefore, we have chosen for the burst pressure studies bTEBVs with an outer size of 6 mm (tube inner diameter of 4 mm) and 1.8 mm (central tube diameter of 0.8 mm). The device used is shown in Fig 6A; for the experiments described here (37°C) the alternating flow rate was adjusted to 3–6 ml/min and the pressure was set between 25 and 100 mbar (18.7 and 75.0 mm Hg). Using this setting the vessels remained intact for over four weeks. During the pulse cycles the diameter of the vessels changed. As an example, the outer diameter of the smaller vessels expanded from 1.8 mm to 2.05 mm (Fig 6B–6E).

**Fig 7:**
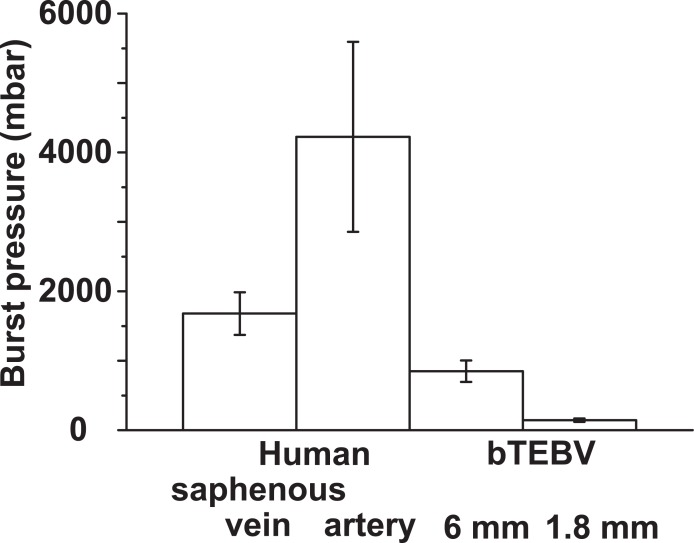
Comparison of the burst pressures of vessels. Physiological human saphenous veins show an average burst pressure of 1680±307 mbar, while the internal mammary arteries measure values between 2031±872 and 4225±1368 mbar. In comparison the bTEBV, fabricated here, display a lower resistance with 850±155 mbar for the larger vessels (of 6 mm) and with 145±24 mbar for the smaller ones (of 1.8 mm). The values for the human human saphenous veins as well as the human internal mammary arteries are taken from the literature [14,73–77].

In order to determine the burst pressure the primary pressure was increased, while the alternative flow rate remained unchanged. Under those conditions the burst pressure for the larger tubes was determined to be 850±155 mbar (n = 20), while that for the smaller 1.8 mm tubes was 145±24 mbar. In order to determine the burst pressure the primary pressure was increased, while the alternative flow rate remained unchanged. Under those conditions the burst pressure for the larger tubes was determined to be 850±155 mbar (n = 20), while that for the smaller 1.8 mm tubes was 145±24 mbar. In our studies the corresponding values for the 6 mm tubes reached already half of it of the published burst pressure of human saphenous veins with 1680±307 mbar [14] and at a wall thickness of ≈ 250 μm [73,74]. In a further comparison, the human internal mammary arteries show a higher resistance and are characterized by a burst pressure of 2031±872 to 4225±1368 mbar [75] at a wall thickness of 350–710 μm [76,77]; Fig 7.

### Determination of the resistance/hardness of the biomaterial

As a parameter for the elastic modulus the hardness of the *N*,*O*-CMC-polyP scaffold was determined. The newly-determined ferruled optical fiber-based nanoindenter was used as outlined under “Material and methods”. The elastic modulus of a biomaterial used for fabrication of the vessel graft should be close to the values measured for physiological cells [78]. The elastic moduli of human arteries and veins are ≈ 455 kPa [79]. In comparison, graft types prepared from PET or ePTFE are considerably harder with a value of 1,900 kPa [80] and 2,200 kPa [81], respectively. The elastic modulus of the vessels, prepared with the *N*,*O*-CMC-based biomaterial, described here, acquired a hardness of 475±61 (n = 10) kPa after an incubation period of 5 min in 2.5% [w/v] CaCl_2_ solution.

**Fig 8:**
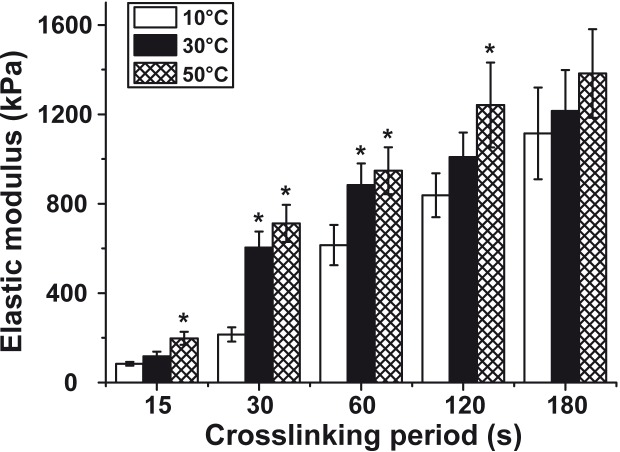
Determination of the elastic modulus of the biomaterial, used for fabrication of the bTEBV. Hardening of the material was performed using 2.5% CaCl_2_ in 70% ethanol. The measurements were performed at 10°C (open bars), 30°C (closed) or 50°C (cross-hatched), using the ferrule-top nanoindenter. The determinations were performed in saline; the moduli are given in kPa. Significant values with respect to the values measured at 15 s and 30°C are marked; * *P* < 0.01.

If 70% ethanol is added to the hardening solution consisting of 2.5% [w/v] aqueous CaCl_2_ the hardening process can be decisively accelerated (Fig 8). Even after a duration of the bTEBV for only 15 s the tubes reach an elastic modulus of 117±21 kPa (at 30°C), a value which increases during an incubation period of 180 s to 1,215±183 kPa. If the hardening process is performed at a lower incubation temperature than 30°C, e.g. at 10°C the hardening process proceeds slower, starting with only 84±9 kPa, but reaching after 180 s almost the same value as measured at 30°C. If the incubation temperature is increased to 50°C the kinetics of the hardening process is very much the same as measured for 30°C (Fig 8).

### Effect of the adhesion-promoting oligo-/polymers on growth of EA.HY926 cells

**Fig 9:**
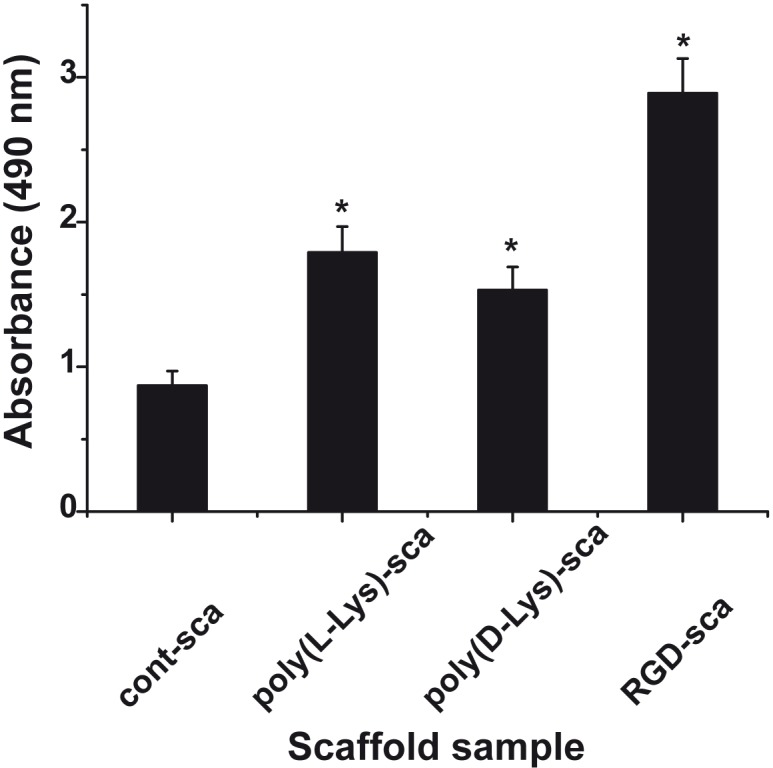
Influence of the different additives to the universal, basic scaffold on cell growth/viability. The scaffold is prepared of *N*,*O*-CMC, silica, polyP and alginate/gelatin in a fixed sequence. The adhesion-promoting oligo/polymers, the polycationic poly-l-lysine and poly-d-lysine as well as His/Gly-tagged RGD were incorporated into the scaffold as described under “Material and methods”. The biomaterial was printed to 0.95 to 1.0 cm discs and placed into 48 well plates. After an incubation period of 7 d the growth/viability of the cells was determined using the XTT assay and the absorbance was measured at 490 nm. Both the “control-scaffold” (cont-sca) and the bioactive scaffolds “poly(l-Lys)-scaffold” (poly(l-Lys)-sca), “poly(d-Lys)-scaffold” (poly(d-Lys)-sca) and “RGD-scaffold” (RGD-sca) were examined. After an incubation period of 7 d the amount of insoluble tetrazolium salt formed was determined. The standard errors of the means (SEM) are indicated (n = 10 experiments); * *P* < 0.05.

The effect of the polycationic poly-l-lysine and poly-d-lysine as well as the His/Gly-tagged RGD on viability/growth of EA.HY926 cells was determined using the XTT assay. The cells, in 48 well plates, were incubated with the following scaffold samples: “control-scaffold”, “poly(l-Lys)-scaffold”, “poly(d-Lys)-scaffold” or “RGD-scaffold”. After an incubation period of 7 d the amount of insoluble tetrazolium salt formed was determined. With respect to the “control-scaffold” all scaffolds supplemented with the adhesion-promoting compounds significantly increased the cell concentration from 0.87±0.11 (“control-scaffold”), to 1.79±0.19 (“poly(l-Lys)-scaffold”), 1.53±0.19 (“poly(d-Lys)-scaffold”) and 2.89±0.24 (“RGD-scaffold”), reflecting that the His/Gly-tagged RGD peptide displays the strongest effect on cell growth (Fig 9).

**Fig 10:**
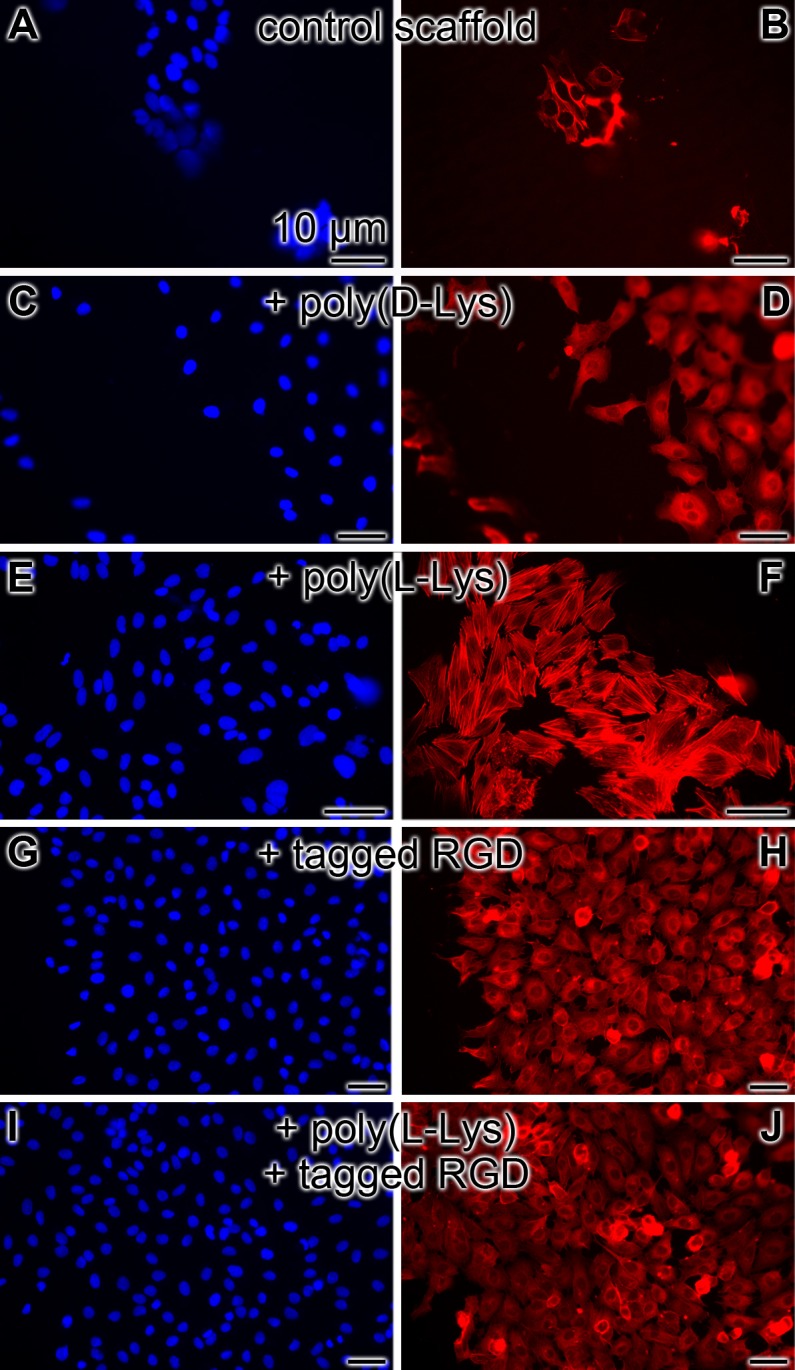
Density of EA.HY926 cells grown on different scaffolds for 2 weeks. **Left panel**: staining with DRAQ5; **right panel**: staining for the cytoskeleton structures, actin; the fluorescent immunostained cells are shown. Density of the cells (**A** and **B**) onto “control-scaffold”, (**C** and **D**) onto “poly(d-Lys)-scaffold”, (**E** and **F**) onto “poly(l-Lys)-scaffold”, (**G** and **H**) onto “RGD-scaffold” and finally (**I** and **J**) onto a scaffold containing both poly(l-Lys) and His/Gly-tagged RGD.

Those cell growth studies were paralleled with studies on the density of cells attached onto the surface of the scaffolds. The different types of scaffolds were incubated with EA.HY926 cells for 2 weeks under conditions described in “Material and methods”. After incubation, the cells attached to the surface of the scaffold, were fixed in formaldehyde vapor and stained for cytoskeleton structures, actin, with antibodies and for DNA in the nucleus with DRAQ5 (Fig 10). The data revealed that only a few clusters of cells are present on the surface of the “control-scaffold” (Fig 10A and 10B; Fig 1B [≈ 25 cells/mm^2^]), while a higher density is seen onto “poly(d-Lys)-scaffold” (Fig 10C and 10D; Fig 1C [≈ 160 cells/mm^2^]), and—even more—onto “poly(l-Lys)-scaffold” (Fig 10E and 10F [≈ 230 cells/mm^2^]). The highest density is seen on scaffold containing the His/Gly-tagged RGD “RGD-scaffold” (Fig 10G and 10H; Fig 1D [≈ 310 cells/mm^2^]). A co-addition of poly(l-Lys) to the His/Gly-tagged RGD-scaffold (poly(l-Lys)-scaffold/RGD-scaffold) is apparently not a better substrate for the EA.HY926 cells to attach to (Fig 10I and 10J).

In order to approach the question if the cells are only loosely attached to the scaffold materials, the assay specimens were subjected to mechanical forces, by vigorous shaking, prior to the fixation of the samples. After such a treatment for 5 min, the cells were fixed and then stained with DRAQ5. No significant differences to the assays that remained without shear stress could be determined. The numbers were found with the “poly(d-Lys)-scaffold” ≈ 145 cells/mm^2^, onto the “poly(l-Lys)-scaffold” ≈ 240 cells/mm^2^, and again with the highest density on the scaffold incorporated with the His/Gly-tagged RGD “RGD-scaffold” ≈ 345 cells/mm^2^. From these results we can concluded that the cells growing onto the scaffolds withstand mechanical stress.

### PolyP effect on clotting time

Under standard determination conditions, described under methods, the effect of the polyP preparations used in the present study was determined in a coagulometer. In the absence of the polymer the clotting time is 345±57 s, after addition of CaCl_2_; this value is not changed significantly if the plasma clotting assay is supplemented with either polyP, complexed with Ca^2+^ (382±62 s), or 10 μg/ml of beads, formed of *N*,*O*-CMC, Na-polyP and hardened with aqueous CaCl_2_ solution (339±49 s).

### Determination of the steady-state-expression of CD31

**Fig 11:**
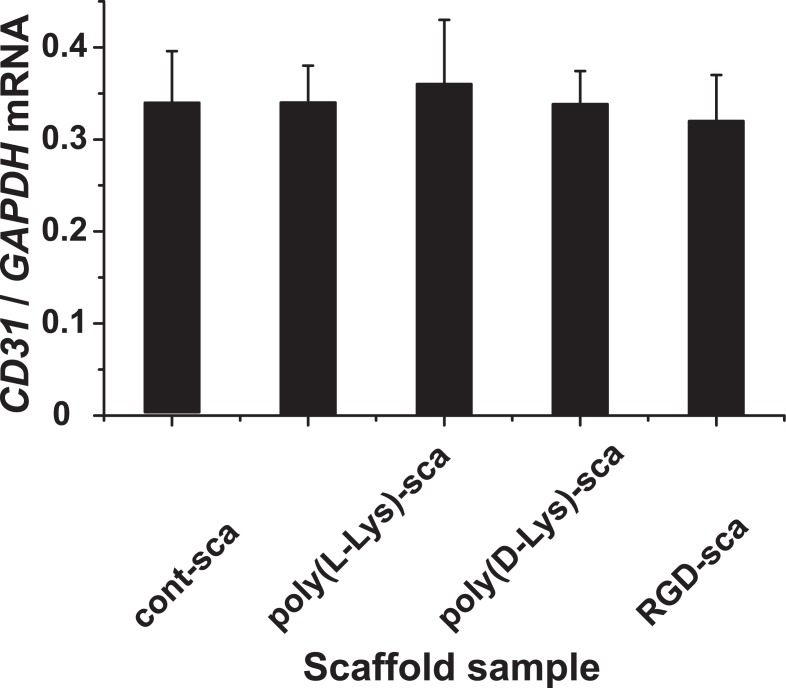
Expression of *CD31* in EA.HY926 cells after cultivation on different scaffolds. The EA.HY926 cells were incubated for 9 d in culture flasks und routine conditions (control), onto “control-scaffold” (cont-scaf), “poly(d-Lys)-scaffold” (poly(d-Lys)-scaf), “poly(l-Lys)-scaffold” (poly(l-Lys)-scaf) and “RGD-scaffold” (RGD-scaf). Then the cells were collected, the RNA isolated and subjected to RT-qPCR. The expression level determined for *CD31* was correlated with the level for *GAPDG*. The means ± SD are shown (5 experiments/time point).

The technique of RT-qPCR was applied to determine the gene expression level for *CD31* in the EA.HY926 cells. After a total incubation period of the cells in normal culture flasks or onto the different scaffolds (“control-scaffold”, “poly(d-Lys)-scaffold”, “poly(l-Lys)-scaffold” and “RGD-scaffold”) for 9 d, the RNA was isolated and the expression level for *CD31* was determined. The values obtained were correlated with the respective expression levels determined for *GAPDH*. The data, summarized in Fig 11, show that in all assays the expression value for *CD 31* varied around 0.34 with respect to the expression of *GAPDH*, indicating that the cells did not change their expression profile, at least with respect to the CD31, a gene which is encoded by the *PECAM1* gene [82]. In parallel, we tested the expression of the gene encoding for the *vascular endothelial-cadherin*, a major endothelial adhesion molecule, controlling cell-cell interaction [83,84]. The steady-state-expression level was found to vary not significantly around a ratio to *GAPDH* of 0.96, irrespectively of the matrices used for the cultivation (data not shown).

## Discussion

**Fig 12:**
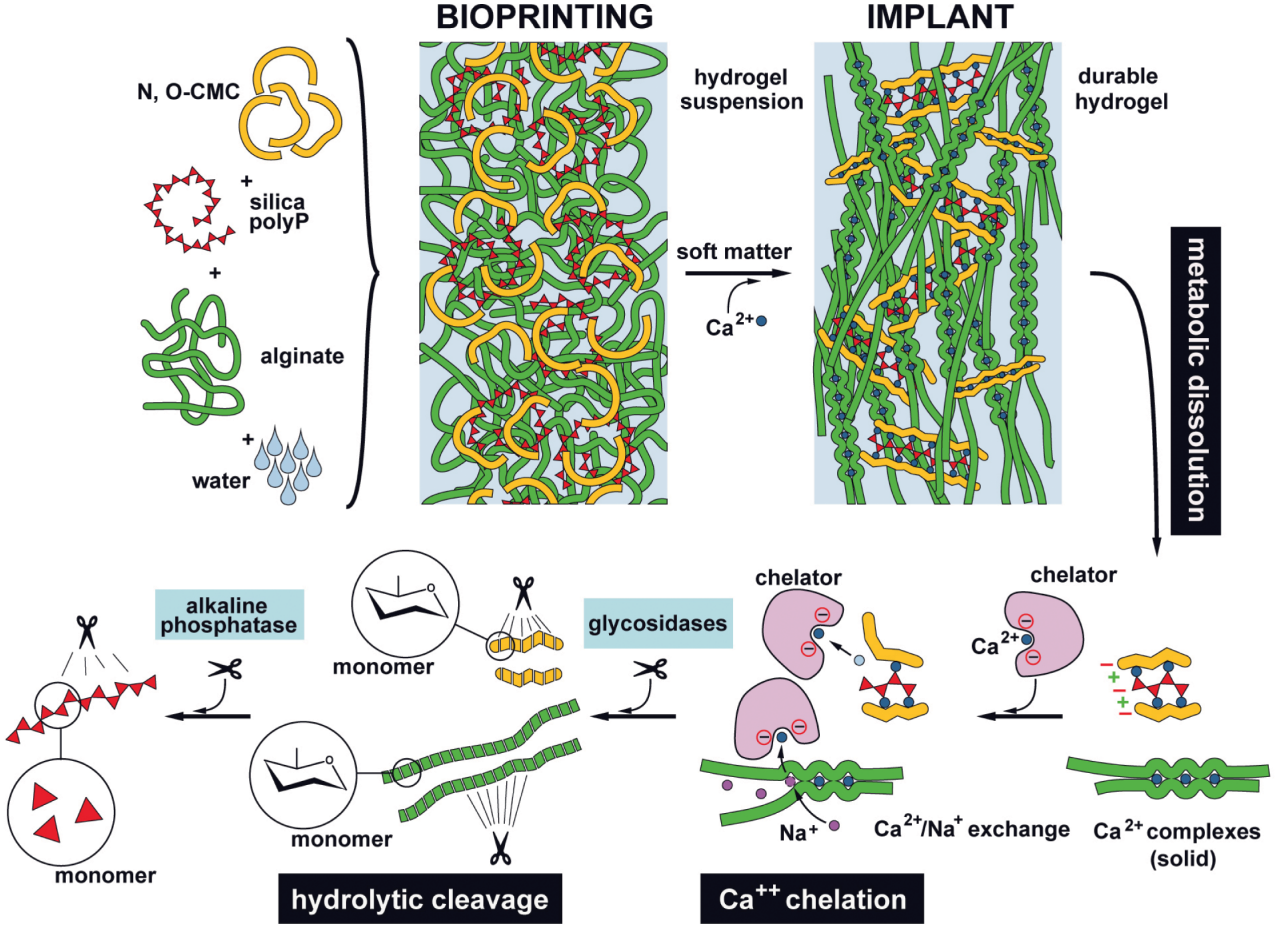
Composition of the polyanionic, printable scaffold material. The basic scaffold of the bioprinted discs or biofabricated vessels (bTEBV) are fabricated by *N*,*O*-CMC, alginate, polyP and silica. Due to ionic linkages the cation Ca^2+^ crosslinks those polymers and forms a more solid implant material. The durable hydrogel formed is proposed to be metabolically dissolved, via glycosidases and alkaline phosphatases (as well as carbonic anhydrases) to the respective monomers. The exchange of Ca^2+^ by Na^+^ is assumed to be mediated by both inorganic and organic chelators.

Since cardiovascular diseases are the leading cause of mortality around the globe, intensive effort has been undertaken to overcome the major hurdles and drawbacks of the hitherto used synthetic grafts, made of PET and ePTFE. There are especially the small diameter graft vessels, with an inner diameter of less than ≈ 8 mm, which are in the center of recent, relevant studies. Instead of these synthetic vessels strong efforts have been undertaken to develop new strategies to develop tissue engineered blood vessels which are biotechnologically fabricated. One promising approach is based on the application of decellularized matrices of natural vessels, since they display the physiological scaffold organization and mechanical properties and are the suitable biological substratum for endothelial cells to adhere to [85,86]. However, traces of antigenic determinants on the material may cause inflammations or other adverse effects, and addition of growth factors, e.g. endothelial growth factor (VEGF), is required to biologize their surfaces [87]. In our approach we used a universal, inert scaffold that is composed of the basic polymeric anionic compounds *N*,*O*-CMC and alginate, enriched with the biologically active polymers silica, polyP and gelatin. This basic material is linked together by the cation Ca^2+^, a process during which the polymers turn from a randomly oriented pattern to a concerted, oriented arrangement [88,89]; Fig 12. During this process the scaffold becomes solid, durable and biologically active. As seen from the growth studies, as well as the cell adhesion behavior, those matrices are suitable for the endothelial cells, we have used EA.HY926 cells, to attach to. The striking advantage of the material described here, the bTEBV, is seen in the fact that its hardness can be adjusted to values existing in arteries and veins, by changing the duration of the CaCl_2_ incubation; even more, the material can be additionally hardened by co-addition of ethanol. By that the bTEBV can be used for a personalized application as an anatomically adequate implant.

In addition the bTEBV can be produced over a wide range of internal and outer diameters, by varying the distance of the center of the central stab to the outer edge of the outlets of the aperture disc or by varying the diameter of the central stab. In particular, small diameter vessels in the size range ≤ 5 mm can be fabricated, e.g. with an outer diameter of 1.8 mm and an inner diameter of 0.8 mm as described here. This size range turned out to be critical with regard to potential thrombotic complications, as reported for synthetic graft materials [16].

Another great advantage of the polyanionic material used for the bTEBV is that, cationic polymers, e.g. poly(l-Lys), poly(d-Lys), and especially His/Gly-tagged RGD can be incorporated. Those additives turn the material to a suitable template for the endothelial cells, since these cells readily attach on those derivatized matrices. This functionalization is expected to prevent the formation of thrombosis after (potential) grafting. A further very favored property of the polymeric materials of the universal scaffold, described here, is that they can be metabolized in humans (Fig 12). The hydrolysis of alginate in human tissue has been described; it has been reported that degradation of alginate-based biomaterials *in vivo* occurs after disintegration of the material in response to a gradual exchange of gelling Ca^2+^ ions with Na^+^ [90]. Likewise, chitosan is metabolically hydrolyzed by macrophages via chitotriosidase [91]. Obvious is the metabolic disintegration of polyP by ALP [92]. Finally biosilica is assessable to the carbonic anhydrases, a family of enzymes that is related to sponge-specific silicase [93]. The removal of Ca^2+^ from the “stabile” polymer complexes within the universal scaffold might be achieved by oxalate chelation [94] as well by organic polymers in the extracellular environment [95]. This property of bTEBV to be prone to metabolic turnover might open the door for an inclusion of the bTEBV in the physiological regeneration of the vessel walls.

## Conclusion

Taken together, the data presented describes a new biomaterial that allows the fabrication of bTEBV. It is based on an universal scaffold that contains a backbone built by two natural polymers, first by alginate, and second a modified chitosan, *N*,*O*-CMC. These two polyanionic polymers can be processed/hardened with Ca^++^ but also with biologically active polymers, e.g. biosilica and polyP, or adhesion-promoting oligomers, like poly(l-Lys) or His/Gly-tagged RGD. We show that this biomaterial is an excellent scaffold to be used for bioprinting and biospinning and for the composition of small diameter blood vessels (diameter < 6 mm) that are urgently needed in clinics for patients needing prosthetic grafts for coronary and peripheral vessels. Those vessels, described here, are custom-made using a home-made extruder, likewise described. It is hoped that those bTEBV are further evaluated and translated into clinical use soon.
